# Ultra sonographic indices of the carotid artery in healthy adult population of southwest, Nigeria: a cross-sectional study

**DOI:** 10.11604/pamj.2023.44.97.30386

**Published:** 2023-02-20

**Authors:** Abiola Omobonike Adekoya, Ayodeji Anike Olatunji, Racheal Adeyanju Akinola, Olatunde Odusan, Adesola Olubunmi Adekoya, Mojisola Adejoke Olusola-Bello, Olatunbosun Oladipupo Olawale

**Affiliations:** 1 Department of Radiology, Olabisi Onabanjo University Teaching Hospital, Sagamu, Nigeria,; 2 Department of Radiology, Lagos State University Teaching Hospital, Ikeja, Nigeria,; 3 Department of Internal Medicine, Endocrine Unit, Olabisi Onabanjo University Teaching Hospital, Sagamu, Nigeria,; 4 Department of Pediatrics, Endocrine Unit, Babcock University Teaching Hospital, Ilisan-Remo, Nigeria,; 5 Department of Chemical Pathology, Olabisi Onabanjo University Teaching Hospital, Sagamu, Nigeria

**Keywords:** Atherosclerosis, carotid flow velocities, subclinical atherosclerosis

## Abstract

**Introduction:**

atherosclerosis develops insidiously, offering time and opportunities for early detection. Screening for subclinical atherosclerosis via structural wall changes and flow velocities among apparently healthy adults using carotid ultrasonography may help its early detection, offer timely intervention and reduce morbidity and mortality.

**Methods:**

a cross-sectional study of 100 participants with a mean age of 56.1 ± 6.9 years, were enrolled from a community population. Both carotid arteries were examined for plaques, carotid intima-media thickness (CIMT), and flow velocities - peak systolic velocity (PSV), end-diastolic velocity (EDV), pulsatility index (PI), and resistive index (RI) using 4-12MHz linear array transducer. Visceral obesity, serum lipids, and blood glucose were also evaluated and correlated with ultrasound findings.

**Results:**

the mean CIMT was 0.07 ± 0.02cm and 15% of the participants had increased CIMT. Statistically significance but weak correlations were observed between CIMT and FBG (r = 0.199, p = 0.047), EDV (r =0.204, p= 0.041), PI (r = -0.287, p = 0.004) and RI (r = -0.268, p =0.007). Statistically significance with modest correlations were observed between EDV and PSV (r = 0.48, p = 0.000), PI (r = -0.635, p = 0.000) and RI (r = -0.637, p = 0.000). The PI and RI showed strong correlation with statistical significance (r= 0.972, p = 0.000).

**Conclusion:**

statistical significance in the flow velocities, derived flow indices and increased CIMT may be an early indication of subclinical atherosclerosis. Therefore, ultrasonography may facilitate its early detection and possible prevention of complications.

## Introduction

Atherosclerosis is a chronic immune-inflammatory fibro-proliferative disease of large and medium-sized arteries of varying etiologies [1]. It is the leading cause of morbidity and mortality in westernized society and the primary cause of cardiovascular diseases (CVD), resulting in myocardial infarction, stroke, and peripheral arterial disease [2-4]. Known risk factors for developing atherosclerotic cardiovascular disease (ASCVD) include age, smoking, diabetes mellitus (DM), hypertension (HTN), sedentary lifestyle, obesity, and deranged lipids [5,6]. These, however, do not accurately predict ASCVD development [6]. Early atherosclerosis is usually asymptomatic and clinically dormant as subclinical atherosclerosis (SCA) [7]. The early structural arterial changes involve smooth muscle cell proliferation, subendothelial macrophage migration, and fatty streak formations with the eventual development of matured vulnerable plaques [7]. This asymptomatic slow disease build-up offers time for screening and detection of these changes in the vascular structures [8].

Ultrasonography (US) evaluation of the carotid artery for SCA has been validated while various other non-invasive methods for its clinical risk assessment exists [9,10]. Coronary artery calcium score (CACS), CIMT, and plaque on carotid artery ultrasound (CU) and ankle-brachial index (ABI) have proven to be useful [11,12]. Computed tomography (CT) CACS better predicts future CVD and coronary events, however, the relatively poor mobility of CT scanners with ionizing radiation and late identification of atherosclerotic lesions are some drawbacks [13,14]. Measurement of ABI, though safe and available, is dependent on the presence of advanced stenotic disease with reduced ABI reported in advanced atherosclerosis [10]. Carotid artery US is a safe, patient-friendly, reproducible, and relatively affordable imaging technique that has been validated for generalized atherosclerosis burden and vascular disease risk [15-17]. Increased CIMT is reportedly associated with future CVS events, while ultrasound-derived carotid plaque burden (CPB) has a similar predictive value as CACS for future CVD event development [18]. Carotid artery structural changes and flow velocities using CU amongst high-risk individuals have been widely reported, however, there is a paucity of such studies on individuals with no known cardiovascular disease risk factors (CVRFs).

The objective of this study was to determine the prevalence of atherosclerosis via structural wall changes and blood flow velocities of the carotid arteries of apparently healthy adults in our environment as this may facilitate their early detection of SCA. We hypothesized that SCA may be absent in them as they have no known CVRFs.

## Methods

**Study design and setting:** this was a hospital-based cross-sectional study approved by our hospital´s Health Research and Ethics Committee (NHREC/08/10/2012). One hundred non-hypertensive, non-diabetic adults aged 30 - 70 years were purposively enrolled for a year (December 2017 - November 2018) from a community population attending the tertiary hospital. All the participants gave informed/written consent before participating in the study.

**Study participants:** all participants were selected from the Medical and General out-patient clinics where they had presented for ailments with no known CVRFs, except age. Excluded from the study were patients with HTN, DM, chronic kidney disease (CKD), past or present history of heart failure, myocardial infarction (MI), coronary heart disease (CHD), cerebrovascular accident, and cancer. Pregnant and lactating women, patients on tracheostomy tubes, and central lines, and those with anatomical constraints like short muscular necks, high bifurcation, and tortuous arteries were exempted from the study.

**Methodology and bias removal:** using a pre-tested interviewer-administered structured questionnaire, information on socio-demography, past medical history, current medications, alcohol use, and smoking were obtained as well as anthropometric, clinical, and laboratory parameters. The same ultrasound machine [4-12MHz linear array (PHILIPS® CLEAR VUE 550, Phillips Healthcare, 2014)] was used for all the participants to exclude errors due to inter-equipment variation. Inter-observer variability errors were minimized by calibration and recalibration of the same equipment before use. Strict hygienic measures such as cleaning equipment before and after each use, washing of hands, and use of latex gloves were adhered to, according to standard operating procedures.

**Study size:** this was by convenience sampling.

**Anthropometry and clinical variables:** body weight (kg) was assessed using a standing weighing scale, (Seca® 755, Hamburg, Germany), and placed on an even, horizontal hard surface. Height (m) was measured standing using a stadiometer, (Seca® 755, Hamburg, Germany) with the head positioned in the Frankfort plane [19]. The body mass index (BMI) in kg/m^2^ was calculated with participants classified as underweight (BMI< 17.8), normal (BMI = 17.8-23.6), overweight (BMI = 23.7 - 26.8), and obese (BMI ≥26.9) in males and underweight (BMI <17.8), normal (BMI = 17.8 - 25.6), overweight (BMI = 25.7 - 28.7) and obese (BMI > 28.8) in females [20]. The waist circumference (WC) was measured following the WHO STEPS protocol [21] and classified as normal (WC <82.99cm), overweight (WC = 83-94.99cm) and obese (WC >95cm) in women, and normal (WC <83.99cm), overweight (WC = 84-95.99cm) and obese (WC >96cm) in men [22]. An average of two brachial blood pressure measurements [systolic blood pressure (SBP) and diastolic blood pressure (DBP)] by auscultation using Accuson® mercury sphygmomanometer with a suitable cuff size in a sitting position, after 5 minutes rest was recorded.

**Laboratory procedure and definitions:** participants' venous blood samples (10mls) were collected aseptically between 07:00 and 08:00 (GMT + 1), after an overnight fast, and assayed for fasting blood glucose (FBG) and serum lipids. Two milliliters (mL) and 3mL of blood were dispensed into fluoride oxalate and plain sample bottles for FBG estimation and lipid assay respectively. Plasma samples for FBG were analyzed immediately on the day of collection, while samples for lipid assay were centrifuged (swing-bucket) within two hours of collection at 3000 revolutions per minute (RPM) for five minutes. The separated serum samples in plain plastic screw-capped containers were stored frozen (-20°C) and assayed for serum lipids within one week of collection. Colorimetric kits manufactured by Randox Laboratories Ltd, United Kingdom, were used for the assay of FBG, Total Cholesterol (TC), Triglyceride (TG), and high-density lipoprotein cholesterol (HDL-C). Low-density cholesterol (LDL-C) was calculated using the Friedewald formula. Laboratory evaluation included FBG (normal 70-110mg/dl) [23] and normal fasting serum lipid (TG<200mg/dl, TC <200mg/dl, LDL-C <160mg/dl and HDL-C >40mg/dl) [24]. All blood samples were analyzed in our hospital´s Research Laboratory by a Consultant Chemical Pathologist.

**Figure 1:**
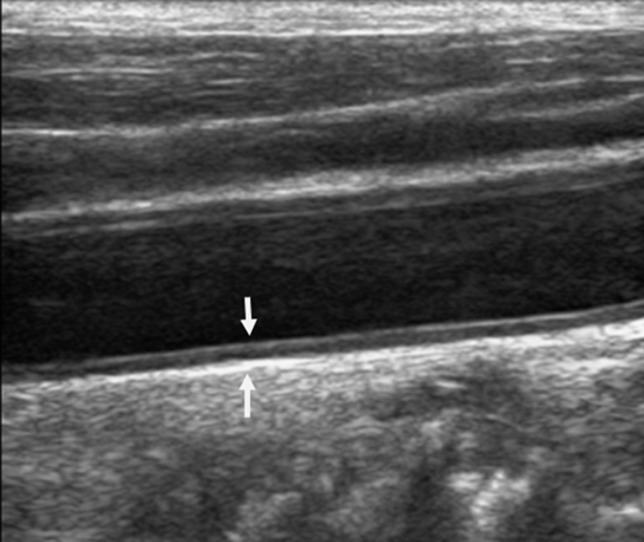
B-mode, longitudinal ultrasound of the carotid artery showing the intima-media thickness in between the white arrows

**Ultrasound evaluation and definition:** the CIMT is the distance between the leading edge of the first hyper-echogenic line (lumen-intima interface) to the leading edge of the second hypo-echogenic line (media-adventitia interface) of the posterior wall of the carotid artery [25]. Using the Mannheim criteria, carotid plaques (CP) are the focal echogenic structures encroaching into the arterial lumen with a thickness of more than 1.4mm when measured from the media-adventitia interface to the intima interface (Figure 1) [25]. Three radiologists with 5-years of experience in CU concurrently performed the US of the participants, each blinded to the result of the other. The participants were placed in a supine position with jewelry and clothing removed from the study area. The chin was raised and slightly extended and the head rotated about 45° away from the examined side after applying the warm water-based ultrasonic gel.

Carotid B-mode, color, and spectral Doppler US in the longitudinal plane of the common carotid artery (CCA), carotid bulb (CB), and internal carotid artery (ICA) on the right and left sides were examined (Figure 1). Three measurements of CIMT, measured with in-built electronic calipers after freezing the images, at the far wall were obtained at the CB, 1cm below and above the bulb for CCA and ICA respectively. Left and right CIMT measurements were averaged and an overall CIMT was taken as a mean of both. Values between 0.9-1.4mm were considered as increased CIMT and >1.4mm indicated atheromatous plaque [26]. The near and far walls of carotid arterial segments were evaluated for CP bilaterally. The echogenicity of CP was classified as isoechoic, hypoechoic, hyperechoic, or calcified, and the surface was expressed as smooth, irregular, or ulcerated. Location, mobility, and the number of arterial segments with plaques were also noted. Spectral waveforms of PSV and EDV were obtained from continuous-wave Doppler examination within one cardiac cycle at an angle of 45 - 600 in the distal CCA and proximal ICA, about 2cm beyond the bifurcation bilaterally. The highest and lowest velocities during systole and diastole were taken as PSV and EDV, respectively. Three measurements taken bilaterally were averaged and overall PSV and EDV were taken as a mean of the CCA and ICA. In cases of atrial fibrillation, average values of five consecutive velocity rhythms were used. Resistive and pulsatility indices which are dependent on the blood-shape waveform and independent of the insolation angle were derived [27]. The pulsatility index (PI), known as the Gosling index, is the difference between PSV and EDV divided by mean flow velocity [28] and RI (Pourcelot index) is the difference between PSV and EDV divided by PSV [27]. The average scan time was 30 minutes per patient. After the procedure, the gel was wiped off the participant´s skin with soft tissue paper, and the neck returned to its normal position.

**Data management and statistical methods:** the generated data were analyzed using the Statistical Package for the Social Sciences (SPSS) for Windows IBM compatible version 23.0. Continuous variables were expressed as mean± standard deviation and categorical variables as proportions and frequencies (percentage). Values were compared for differences using the Student´s t-test and Chi-square tests. Analysis of Variance (ANOVA) and Pearson correlation coefficient (r) were used to determine the relationship between continuous variables with statistical significance set at p< 0.05.

## Results

One hundred volunteered eligible participants completed the study and were analyzed. Sixty-two females and 38 males (male-to-female ratio of 0.6: 1) with a mean age of 56.1 ± 6.9 years participated in the study. There were 21 (21%), 48 (48%), and 31 (31%) in the < 50 years, 51-60 years, and 61-70 years age groups respectively. The socio-demographic parameters of study participants are shown in Table 1. Their mean FBG was 79.9 ± 13.1mg/dl with mean SBP and DBP at 119.6 ± 11.5mmHg and 77.5 ± 8.7mmHg, respectively. There were no statistical significances in the mean WC (t = 0.038, p = 0.970), BMI (t = 0.046, p = 0.963), FBG (t = -1.211, p = 0.229), LDL -C (t = -0.705, p = 0.482), HDL -C (t = -0.944, p = 0.348), TG (t = 1.811, p = 0.073), and TC (t = -0.926, p = 0.357) along gender divide (Table 2).

**Table 1:** socio-demographics of the study population

Variables	Frequency; n = 100 (%)
**Marital status**	
Single	11%
Married	75%
Separated/divorced	8%
Widow	6%
**Educational status**	
None	3%
Primary	18%
Secondary	36%
Post-secondary	43%
**Occupation**	
Unemployment	8%
Artisan	12%
Trading	35%
Civil service	35%
Business	7%
Retired	3%
**History of smoking**	
Non-smoker	96%
Ex-smoker	3%
Current smoker	1%
**History of alcohol consumption**	
Non-consumer	85%
Ex-consumer	10%
Current consumer	5%
**History of physical activity**	
None	71%
< 1 -2 times/week	15%
>3 times/week	14%

**Table 2:** mean and proportional distribution of clinical and lab parameters along the gender divide

Variables	Mean ± SD	Male; n (%)	Female; n (%)	X^2^	P-value
**BMI (kg/m^2^)**	27.13 ± 6.12			0.592	0.744
Normal		17 (44.7)	24 (38.7)		
Overweight		13 (34.2)	21 (33.9)		
Obese		8 (21.1)	17 (27.4)		
**WC (m)**	88.47 ± 13.00			0.010	0.995
Normal		14 (36.8)	23 (37.1)		
Overweight		12 (31.6)	20 (32.3)		
Obese		12 (31.6)	19 (30.6)		
**TC (mg/dl)**	205.99 ± 98.18			4.687	0.096
Normal		21 (55.3)	33 (53.2)		
Intermediate		8 (21.1)	5 (8.1)		
High		9 (23.7	24 (38.7)		
**TG (mg/dl)**	105.93 ± 48.05			2.298	0.130
Normal		22 (57.9)	45 (72.6)		
Abnormal		16 (42.1)	17 (27.4)		
**LDL-C (mg/dl)**	153.19 ± 48.05			0.161	0.689
Normal		15 (39.5)	27 (43.5)		
Abnormal		23 (60.5)	35 (56.5)		
**HDL-C (mg/dl)**	34.28 ± 18.67			0.000	0.992
Normal		30 (78.9)	49 (79)		
Abnormal		8 (21.1)	13 (21		

BMI = Body mass index, WC=Waist circumference, TC = Total cholesterol, TG = Triglyceride, LDL-C = Low density lipoprotein cholesterol, HDL-C = High density lipoprotein cholesterol

The mean value of CIMT and flow velocities with their derived values are shown in Table 3. The mean CIMT was 0.07 ± 0.02cm in both gender and 0.07 ± 0.02cm for the age groups <50, 51-60, and 61-70 years with no statistical significance (p = 0.576). The carotid bulb had the highest CIMT bilaterally (0.08 ± 0.03cm). Fifteen percent of the study participants had increased CIMT distributed as 29.5%, 57.4%, and 13.1% at the CCA, bulb, and ICA respectively. The increased CIMT showed no statistically significant differences with almost equal gender distribution (46.7% males and 53.3% females). The mean CIMT had positive weak correlations with statistical significances with FBG (r = 0.199, p = 0.047) and SBP (r = 0.224, p = 0.025). There were no statistically significant differences with BMI (p = 0.987), WC (p = 0.406), TC (p = 0.193), TG (p = 0.532), HDL-C (p = 0.429), and LDL-C (p = 0.126). Carotid plaque seen in 2% of the population (females in the 5^th^ and 6^th^ decades), had equal distribution at the bulbs. They were non-mobile, echogenic, and heterogenic on the right and left respectively with smooth outlines.

**Table 3:** mean value of ultrasonographic parameters of the study population along the gender line

Variables	Gender	N	Mean ± Sd	t-test value	p-value
CIMT (cm)	Male	38	0.71 ± 0.024		
	Female	62	0.71 ± 0.021	-0.125	0.901
PSV (cm/s)	Male	38	78.16 ± 15.96		
	Female	62	76.69 ± 17.98	0.414	0.680
EDV (cm/s)	Male	38	22.60 ± 5.25		
	Female	62	23.79 ± 6.45	-0.966	0.337
RI	Male	38	0.70 ± 0.06		
	Female	62	0.68 ± 0.07	1.695	0.093
PI	Male	38	0.59 ± 0.07		
	Female	62	0.57 ± 0.08	1.685	0.138

CIMT = Carotid intima media thickness, PSV = Peak systolic velocity, EDV = End diastolic velocity, RI = Resistive index, PI = Pulsatility index

The mean flow velocity values were higher on the left and showed statistical significance with moderate positive correlations (r =0.639, p < 0.005). A higher PSV and EDV value was observed in males and females respectively, however, gender comparison showed no statistically significant difference (Table 3). Statistically, significances were observed between CCA-PSV and BMI (p = 0.008), WC (p = 0.017), SBP (p = 0.0008), and DBP (p = 0.019); ICA-PSV and BMI (p =0.0010, WC (p= 0.009), SBP (p = 0.023) and DBP (p= 0.009); CCA-EDV and CIMT (p =0.047), TC (p = 0.004) and LDL-C (p = 0.006) and ICA-EDV and FBG (p = 0.032). The mean PSV showed a weak negative association with BMI, WC, SBP, and DBP with statistically significant differences (p < 0.004) (Table 4). The mean PI and RI were higher in the males with no statistically significant difference (p = 0.138 and 0.093 for PI and RI respectively). Moderate and positive correlations were observed between the right and left PI and RI with statistical significance (p < 0.05). However, a strong positive correlation was observed between PI and RI with statistical significance (r = 0.972, p = 0.000). Details of correlations of CIMT, PSV, EDV, PI, and RI with other parameters of the participants are shown in Table 4.

**Table 4:** correlation coefficients between traditional risk factors and carotid intima-media thickness, peak systolic velocity, end-diastolic velocity, pulsatility index, and resistance index of participants

Variables	CIMT; r (P)	PSV; r (P)	EDV; r (P)	PI; r (P)	RI; r (P)
Age (years)	0.081(0.425)	0.013(0.898)	0.051(0.612)	-0.026(0.707)	-0.046(0.649)
WC (cm)	0.181(0.072)	-0.294(0.003)*	-0.144(0.154)	-0.095(0.345)	-0.085(0.402)
BMI (kg/m^2^)	0.164(0.103)	-0.340(0.001)*	-0.147(0.145)	-0.143(0.157)	-0.120(0.233)
TG (mg/dl)	0.179(0.075)	-0.101(0.317)	-0.102(0.314)	0.047(0.640)	0.062(0.537)
TC (mg/dl)	-0.171(0.090)	-0.190(0.058)	-0.131(0.195)	-0.017(0.864)	-0.036(0.724)
LDL-C (mg/dl)	-0.123(0.224)	-0.179(0.075)	-0.120(0.234)	-0.019(0.853)	-0.033(0.748)
HDL-C (mg/dl)	-0.173(0.085)	-0.052(0.605)	0.026(0.795)	-0.103(0.308)	-0.119(0.237)
CIMT (cm)	-	-0.091(0.366)	0.204(0.041)*	-0.287(0.004)*	-0.268(0.007)*
PSV (cm/s)	-0.091(0.366)	-	0.480(0.000)*	0.343(0.000)*	0.268(0.007)*
EDV (cm/s)	0.204(0.041) *	0.480(0.000)*		-0.635(0.000)*	-0.637(0.000)*
PI	-0.287(0.004)*	0.343(0.000)*	-0.635(0.000)*		0.972(0.000)*
RI	-0.268(0.007)*	0.268(0.007)*	-0.637(0.000)*	0.972(0.000)*	
SBP (mmHg)	0.224(0.025)*	-0.294(0.003)*	-0.131(0.193)	-0.084(0.409)	-0.075(0.460)
DBP(mmHg)	0.057(0.572)	-0.292(0.003)*	-0.105(0.297)	-0.162(0.107)	-0.141(0.161)
FBG (mg/dl)	0.199(0.047)*	-0.014(0.893)	0.168(0.096)	-0.120(0.235)	-0.119(0.239)

WC = Waist circumference, BMI = Body mass index, TC = Total cholesterol, LDL–C= Low density lipoprotein cholesterol, HDL - C= High density lipoprotein cholesterol, TG = Triglycerides, CIMT = Carotid intima media thickness, PSV= Peak systolic velocity, EDV= End diastolic velocity, PI = Pulsatility index, RI = Resistance index, SBP = Systolic blood pressure, DBP = Diastolic blood pressure, FBG = Fasting blood glucose

## Discussion

This study presents carotid artery ultrasonography (CU) findings in otherwise healthy adults, with previously unreported CVRFs. The CVRFs among the study participants were age, with 79% of the participants older than 50 years, central obesity (31%), generalized obesity (25%), and dyslipidemia (58%). These findings were like earlier reports amongst Nigerian adults in cross-sectional hospital-based studies which reported obesity at 34.2% and 44.7%, dyslipidemia at 68.5% and 44.7%, and hypertension at 49.4% and 40.8% respectively [29,30].

**Carotid intima-media thickness and plaque:** the mean CIMT in this study was similarly reported by Baba *et al*. [31]. However, its value is higher than that reported in Turkey [32] and lower than the controls of Koc *et al*. [33]. The differences could be due to ethnic or racial differences, anatomical selection sites and the instrument used. The CIMT at the CB being significantly higher than the CCA and ICA as similarly reported by Baba *et al*. [31] may be due to anatomy at carotid artery bifurcation and resultant hemodynamic changes [34]. The increased CIMT observed in our study participants was lower than 53.7% and 36.5% reported by Omisore *et al*. [29] and Okeahialam *et al*. [35] respectively. They used varying cutoffs with the presence of CVRFs in their study population. In our study, 2% of the participants had CP which contradicts other studies [29,36] reporting a higher prevalence of CP with statistical significance and a strong positive correlation with serum lipids although known CVRFs were also present [29,36]. A study in China reported significant carotid atherosclerotic plaque formation, independent of other vascular risk factors in pre-hypertensives with the same severity as in hypertensives [37].

**Carotid flow velocities:** there are reports associating carotid flow velocities and CVD in high-risk individuals [38-40], although, its clinical application for vascular complication prevention in people with no known risk factor is limited while flow changes may be present in them. Non-invasive tracking of changes in flow velocity using ultrasonography may provide additional markers for SCA leading to early detection and prevention of atherosclerosis. The PSV and EDV are independently associated with CVD development [38]. The EDV with the capacity of intracranial circulation blood provision at diastole [38], improves future prediction of CVD more than PSV [39] and for ischemic stroke than RI and PSV [38]. In this study, the PSV and EDV values were similar to the normotensives reported by Park *et al*. [40], although they reported higher values in the pre-hypertensive and controls, a difference which could be attributed to co-morbid factors in their study population [40,41]. An earlier report from a case-control study observed an association of high ischemic stroke risk with low CCA EDV while low EDV in hypertensive resulted in decreased arterial dispensability and low diastolic perfusion pressure, leading to low shear stress, hence, atherosclerosis [42,43]. In our study, a moderate correlation was observed between the EDV and PSV with a statistically significant difference.

**Derived carotid flow indices:** pulsatility and resistive indices assess the arterial wall shear stress, an important predictor of cardiovascular risk and atherosclerosis development [44]. The PI reflects the transmission of pulsatile energy into the CVS and end-organ microcirculation while the RI mirrors the vascular flow resistance in the end-organ vessels [40,44]. High carotid flow PI and RI are associated with CVRFs and suggest arterial wall changes [39,41]. In contrast to other studies [38-41], lower PI and RI were observed in this study with a strong positive correlation between the PI and RI. A high PI (>1.60) reported amongst the elderly with high FBG, large WC, and low HDL-C was associated with a higher incidence of stroke with a high hazard stroke ratio [41]. In Taiwan, RI > 0.76 was positively associated with CVD than a lower value [39]. Therefore, the US evaluation of these indices may be helpful in distal arterial vessel resistance and elasticity assessment where they are not easily accessible with ultrasonography.

**Carotid flow velocities, carotid intima-media thickness, and metabolic disorder:** hypertension, hyperglycemia, and dyslipidemia are risk factors for atherosclerotic vasculopathy with a known association with visceral adiposity [36]. A greater association of WC with metabolic syndrome than BMI or body fat percentage (BFP) has been reported. Visceral adiposity via WC is reportedly a better index for CVD development than regional or generalized obesity [45-47]. High WC independently contributes to high levels of TG and low plasma HDL-C concentrations in the body [48]. In this study, significant differences were noted between WC and TC and TG, a finding consistent with Ishihara *et al*. [49]. Weak associations observed between CIMT and WC, BMI, FBG, SBP, DBP, and serum lipids in this study, showed statistical significance only with FBG and SBP. The PSV also showed a weak association with SBP, DBP, WC, and BMI with statistical significance, indicating a relationship between flow velocity and atherosclerotic risk factors.

**Limitations:** a major limitation of this study is that it was hospital-based. This may prevent the actual representation of the findings among the general population. In addition, the carotid wall diameter and echocardiographic parameters of study participants were beyond the scope of this study and hence not evaluated. However, the statistically significant differences in participants with higher CIMT and strong positive correlations between PI and RI are important.

## Conclusion

The statistically significant differences observed in CIMT, flow velocities, and the derived flow indices of participants with no known CVDRFs are essential. Therefore, the combination of structural arterial wall changes and velocity flow rates could be predictive of early diagnosis of SCA and promptly institute preventive measures for atherosclerosis.

### What is known about this topic

- There are widely reported values of CIMT in various individuals with co-morbid disease conditions;
- A CIMT value greater than 0.09mm reportedly connotes atherosclerosis in the co-morbid states;
- Atherosclerotic vessels lose their elasticity and recoil, therefore, increasing their blood flow velocities.

### What this study adds

- Despite various reported carotid US evaluations of different individuals, there is no information on apparently healthy individuals with no known CVDRs which may be a pointer to early detection of non-symptomatic atherosclerosis;
- Statistically significant differences seen between the CIMT, and flow velocities and the derived PI and RI of this study population are reflective of ongoing SCA; in addition, this study provides reference values of CIMT, PSV, EDV, PI, and RI in individuals in our environment with no known CVDRFs;
- The absence of known CVDRFs in people does not exclude them from developing atherosclerosis; therefore, screening of the carotid arteries via US may provide an avenue for early detection of SCA and prompt intervention, reducing morbidity and mortality.
